# Thermal explosion in oscillating ambient conditions

**DOI:** 10.1038/srep29730

**Published:** 2016-07-22

**Authors:** Vasily Novozhilov

**Affiliations:** 1 Centre for Environmental Safety and Risk Engineering, Victoria University, Werribee VIC 3030, Australia

## Abstract

Thermal explosion problem for a medium with oscillating ambient temperature at its boundaries is considered. This is a new problem in thermal explosion theory, not previously considered in a distributed system formulation, but important for combustion and fire science. It describes autoignition of wide range of fires (such as but not limited to piles of biosolids and other organic matter; storages of munitions, explosives, propellants) subjected to temperature variations, such as seasonal or day/night variation. The problem is considered in formulation adopted in classical studies of thermal explosion. Critical conditions are determined by frequency and amplitude of ambient temperature oscillations, as well as by a number of other parameters. Effects of all the parameters on critical conditions are quantified. Results are presented for the case of planar symmetry. Development of thermal explosion in time is also considered, and a new type of unsteady thermal explosion development is discovered where thermal runaway occurs after several periods of temperature oscillations within the medium.

Thermal explosion (known also as thermal runaway or autoignition) is a fundamental combustion science concept describing wide range of observed phenomena. Its importance in understanding fires is that it describes autoignition of various configurations of solid materials such as biomass or refuse- derived fuels, piles of food grains, coal, wood chips, bagasse and compost, as well as storages containing military munitions, solid propellants, pyrotechnics or similar substances. Hazards of these fire scenarios are substantial and well documented in the literature^1^,^2^,^3^,^4^,^5^,^6^,^7^,^8^.

Thermal Explosion theory has been an area of active research for over 80 years. In 1928 in the fundamental paper^9^ Semenov derived, for the first time, critical conditions for thermal explosion. His theory essentially considers a steady-state problem and neglects spatial temperature distribution (zero-dimensional approximation). Later, Frank-Kamenetskii^10^ introduced heat transfer equation with non-linear source and considered temperature distribution in the reaction zone.

Since then thermal explosion theory development has taken different avenues. Some of the most important developments are summarized in the monographs and papers^11^,^12^,^13^,^14^,^15^,^16^,^17^,^18^,^19^,^20^,^21^,^22^. The two major approaches, kinetic and thermal, have been exploited. The first deals essentially with the influence of complicated chemical kinetics, such as different kinetic mechanisms, autocatalysis, parallel reactions and other effects. The second direction considers complicated forms of heat transfer within reacting mixture, as well as between the mixture and its surroundings.

One of the most important problems that have been considered along the second route is thermal explosion under linearly increasing ambient temperature^14^. It is quite natural to consider a problem with oscillating ambient conditions. Motivations for such a problem can be easily found considering thermal explosion in the systems indicated above^1^,^2^,^3^,^4^,^5^,^6^,^7^,^8^. These systems are likely to be subjected to temperature variations such as day/night variations (in regions close to deserts these are especially significant), seasonal (yearly) variations and weather variations. Such variations may cause reacting medium to deviate from thermal equilibrium conditions and develop thermal explosion leading to fires.

The described problem certainly looks very classical, yet it has not been consistently addressed so far. It is evident that variations with very different frequencies and amplitudes need be considered.

For convenience, throughout the paper waved variables refer to dimensional quantities, while unwaved refer to non-dimensional ones.

Major conclusions derived considering the problem with linearly increasing ambient temperature^14^ are as follows. Under low values of the temperature growth rate 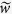 reactive medium temperature increases slowly, and eventually self-acceleration of the chemical reaction is compensated by kinetic deceleration (due to reactants consumption). An exclusion is the case of reaction of the zero order which always leads to thermal explosion for any positive values of the ambient temperature growth rate.

As the ambient temperature growth rate increases, it attains certain critical value 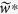 such that for 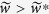
kinetic factors cannot prevent thermal explosion. In contrast to the basic Semenov thermal explosion problem^9^ where critical conditions are determined by the balance between self-acceleration of the chemical reaction and the rate of heat dissipation, in the case of dynamic regimes with monotonically increasing ambient temperature existence of critical conditions is mostly due to reactant consumption (i.e. kinetic factors).

It is clear from this qualitative consideration that if one considers a certain value 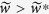 and take a temperature grow profile that deviates (becomes slower) from the linear 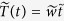 at sufficiently large values of time, then explosion will occur for such a type of the profile. Behaviour of ambient temperature after the time of explosion is irrelevant and consequently temperature 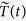 may decrease starting from some point after explosion. These simple considerations show that thermal explosion may occur under oscillating ambient temperature conditions. It is also obvious that if the system is in the state of thermal equilibrium (i.e. in the state below critical for the static ambient conditions) then sufficiently small oscillations cannot lead to thermal explosion. Therefore, critical conditions for thermal explosion in the case of oscillating ambient temperature do exist. Similarly to the static ambient conditions case, and in contrast to the case of linear ambient temperature growth, these conditions are predominantly related to the rate of heat dissipation from the system.

Similar problem was considered in the papers^23^,^24^ but only in “zero-dimensional” formulation, that is neglecting temperature variations across reacting media. This formulation is not physically realistic. In relevant practical applications temperature variations across the medium during self-ignition are significant^25^ and therefore the model of the papers^23^,^24^ is not physically relevant. It does not allow the influence of parameters controlling heat exchange with surroundings, such as Biot number *Bi*, on critical conditions to be investigated.

Mathematically, derivation of the “zero-dimensional” model in^23^,^24^ is inconsistent as it is being obtained by spatially averaging the heat transfer equation with the boundary condition of the first kind at the outer boundary. In the present problem, such a condition implies *Bi* ≥ 1 (intensive heat exchange with surroundings), which is emphasized in^23^. On the other hand, justification of the spatial temperature averaging requires *Bi *≤ 1. In any case, straightforward averaging of the Arrhenius reactive term undertaken in^23^ is widely known in the combustion literature as being unacceptable.

Besides, no estimation of parameters of practical systems are presented in^23^,^24^ to judge whether thermal explosion regimes with oscillating boundary conditions are relevant for practical applications. Since the model is oversimplified and estimations of parameters relevant for practical systems are not provided, papers^23^,^24^ do not prove, in fact, that predictions made in these papers may occur in reality.

The present paper takes into account spatial variation of the temperature, that is considers one-dimensional unsteady model, without further averaging or any other assumptions. It provides systematic investigation of the influence of all the relevant parameters. Both first and second kind boundary conditions are considered. Estimations of parameters relevant to real systems are provided and clearly demonstrate that all the presented phenomena may be observed in real combustion systems.

In the present paper the problem is considered in its most fundamental form in order to achieve major understanding of the process and to evaluate effects of the key parameters. It is always instructive to start with this approach before various complicating issues related to description of particular practical systems are considered. This builds a framework for interpreting behaviours of more complicated systems, and also allows the generalization of the results to be made.

## Mathematical Model

The formulation follows well established scaling procedure^14^ for thermal explosion problems. It is briefly reminded here.

The following scales are used: 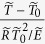 for temperature, 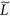 (characteristic size of the vessel) for spatial scale; 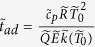 (adiabatic time scale) for time. The parameters have clear physical meaning of 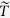 for temperature, 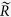
for universal gas constant, 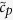 for specific heat, 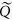 for heat of combustion, 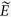 for activation energy. Mean value of the oscillating ambient temperature is indicated by the subscript “0”, and the reaction rate constant at this mean value is denoted by 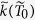.

Reactants consumption is neglected in the present formulation, that is the reaction of the zero order is considered. As explained in the introduction, critical conditions for the problem under consideration are mostly determined by thermal exchange conditions.

As in the classical thermal explosion theory, the symmetrical cases, with the symmetry index *m* = 0 (planar symmetry), *m* = 1 (cylindrical symmetry) or *m* = 2 (spherical symmetry) are considered.

Upon scaling of the heat transfer equation, it is obtained in the non-dimensional form


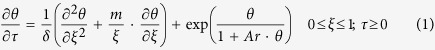


where *θ* is temperature, *τ* is time, *ξ* is spatial coordinate, 
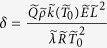
 is Frank-Kamenetskii parameter (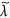 is thermal conductivity), and 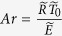 is Arrhenius number.

Due to space limitation, only the results for the planar symmetry case *m* = 0 are presented in the paper. This case describes a slab of material between the two parallel planes where the temperature only varies in the direction normal to the planes.

The two types of boundary conditions (BC) that are sensible from the physical point of view are those of the first kind


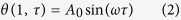


and of the second kind (Newtonian heat exchange)


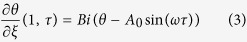


where 
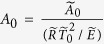
 and 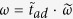 are non-dimensional amplitude and frequency of the temperature oscillations, respectively, *Bi* is Biot number.

At the symmetry plane in the middle of the slab


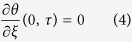


Behaviour of the system is described by the following set of parameters. First of them is *δ* (Frank-Kamenetskii parameter). This is supposed to have subcritical value for the static boundary conditions (*A*_0_ = 0), and describes the degree of subcriticality of the system, i.e. how much lower is the actual parameter *δ* compared to its critical value *δ*_*cr*_ at static ambient conditions.

Other parameters are Arrhenius number *Ar*, and in case of boundary conditions of the second kind, also Biot number *Bi*.

Therefore, there are the following sets of controlling parameters:

(*δ*, *Ar*)- for the boundary conditions of the first kind;

(*δ*, *Bi, Ar*)- for the boundary conditions of the second kind.

**Figure 1:**
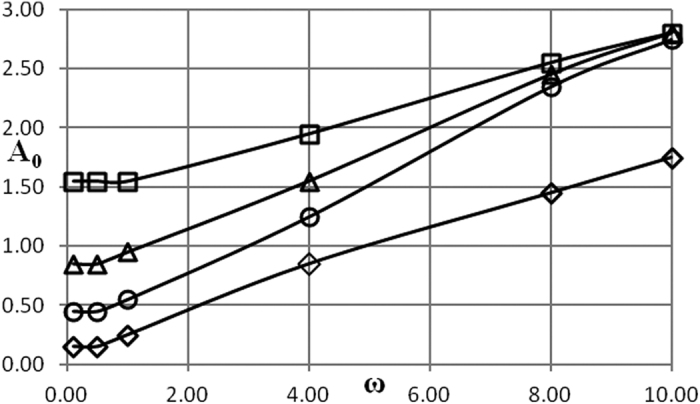
Critical dependencies *A*_0_(*ω*). BC of the first kind. *Ar* = 0 □ − *δ* = 0.2; Δ − *δ* = 0.4; Ο − *δ* = 0.6; ◇ − *δ* = 0.8.

**Figure 2:**
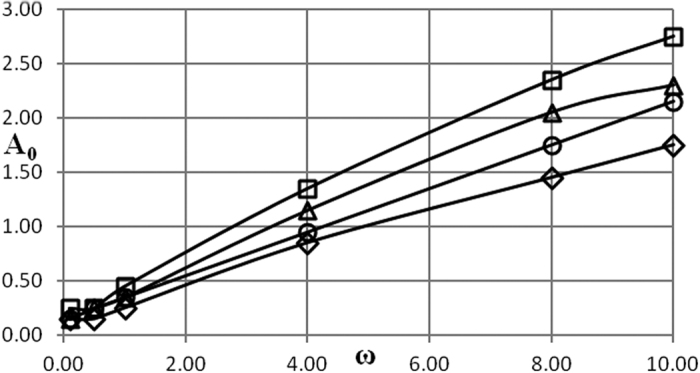
Critical dependencies *A*_0_(*ω*). BC of the first kind. *δ* = 0.8 □ − *Ar* = 0.06; Δ − *Ar* = 0.04; Ο − *Ar* = 0.025; ◇ − *Ar* = 0.0.

**Figure 3:**
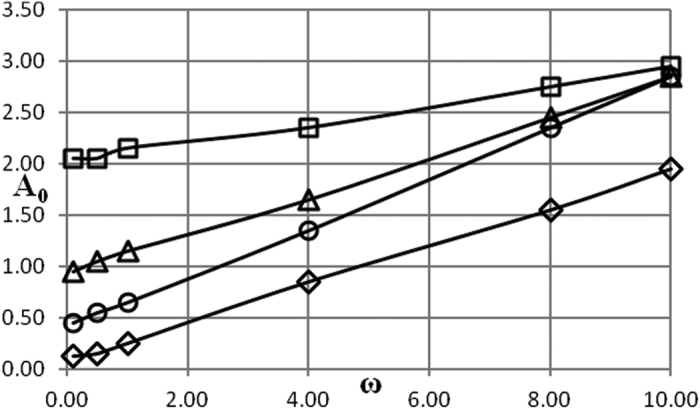
Critical dependencies *A*_0_(*ω*). BC of the second kind. *Ar* = 0; *Bi* = 15 □ − *δ* = 0.1; Δ − *δ* = 0.3; Ο − *δ* = 0.5; ◇ − *δ* = 0.7.

**Figure 4:**
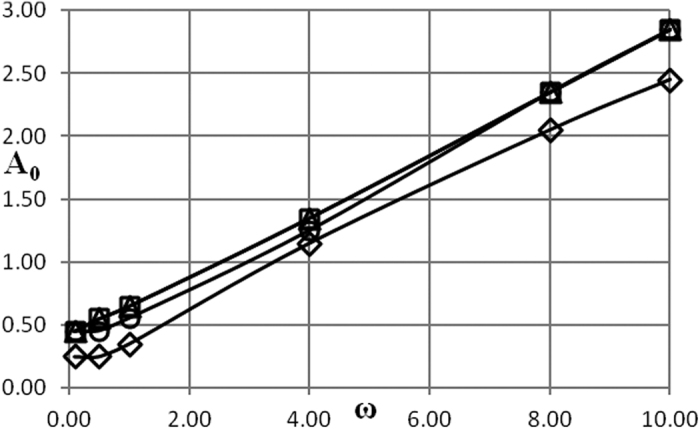
Critical dependencies *A*_0_(*ω*). BC of the second kind. *Ar* = 0; *δ* = 0.5 □ − *Bi* = 20; Δ − *Bi* = 15; Ο − *Bi* = 10; ◇ − *Bi* = 5.

**Figure 5:**
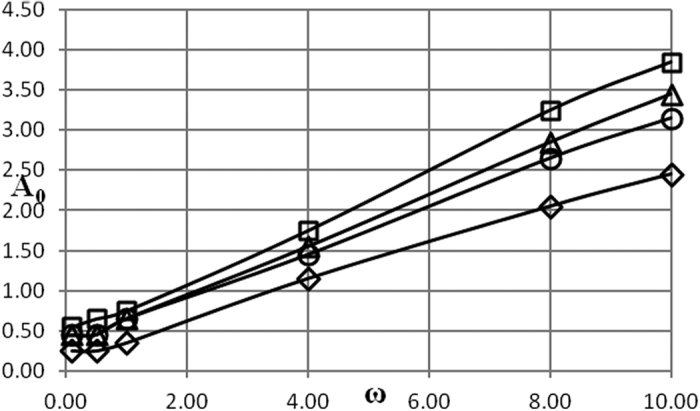
Critical dependencies *A*_0_(*ω*). BC of the second kind. *δ* = 0.5; *Bi* = 10 □ − *Ar* = 0.06; Δ − *Ar* = 0.04; Ο − *Ar* = 0.025; ◇ − *Ar* = 0.0.

Critical conditions are presented in Figs 1, 2, 3, 4, 5 as dependencies *A*_0_(*ω*) at critical boundary with all the other parameters fixed. It is reminded again that, in accordance with the notation convention in the present paper, the both variables *A*_0_ and *ω* (unwaved!) in Figs 1, 2, 3, 4, 5 are non-dimensional.

Equation (1) is solved numerically using Crank-Nicolson scheme with a central difference approximation for the second derivative (only *m* = 0 is considered). To ensure appropriate level of grid-independence, spatial and time steps are refined to the point where relative error in output values does not exceed 10^−4^.

In order to establish the critical boundary *A*_0_(*ω*), infinitely growing solutions need be separated from bounded solutions. In the present non-linear problem this can only be done numerically, and only within a certain degree of accuracy.

The procedure followed in the present paper is as follows. Infinitely growing temperature history profiles (that corresponds to explosion conditions) have an inflection point. Non-dimensional time interval [0, τ^*^] is fixed and relevant parameters are being varied, until an inflection point appears at *τ* = τ^*^. So chosen set of parameters provides an estimate for the exact position of the critical boundary. To limit the dependence of this estimate on the actual value of τ^*^, another estimation is obtained using the interval [0, 2τ^*^]. The parameter τ^*^ is increased until the relative error between the two estimates is within 10%. Then parameters corresponding to the [0, 2τ^*^] estimate are reported as a position of the critical boundary. In the present study, a typical τ^*^ value required to obtain the data presented in Figs 1, 2, 3, 4, 5 is about 100.

## Results and Discussion

### Boundary condition of the first kind

There are two controlling parameters, except for the amplitude and frequency, in this case. These are Frank-Kamenetskii parameter and Arrhenius number. Dependence of critical conditions on parameters *δ* and *Ar* is illustrated in Figs 1 and 2. Figure 1 presents data for different values of the Frank-Kamenetskii parameter that are below the critical one for the constant unperturbed ambient temperature *A*_0_ = 0. The critical value of this parameter for the latter case is, as it is well known^11^, *δ*_*cr*_ = 0.88. The Arrhenius number is fixed in this plot.

It is evident from Fig. 1 that, except for the range of low frequencies 0 < *ω* ≤ ≈ 1 critical dependencies of the amplitude on the frequency are very close to linear.

Figure 1 demonstrates essential feature of the problem considered in the present paper: the system that is thermally stable under constant ambient conditions, may develop instabilities (thermal explosion) if ambient conditions vary in oscillating manner.

Figure 2 demonstrates the effect of Arrhenius number on critical dependencies *A*_0_(*ω*) when *δ* is fixed. Again, all the dependencies shown in Fig. 2 are essentially linear.

Critical amplitude decreases (at fixed frequency) with the decrease of Arrhenius number as it should be considering the form of the chemical reaction term in equation (1).

As it is evident from Figs 1 and 2 nonlinear behaviour of the critical boundary *A*_0_(*ω*) is only observed at very low frequencies, that is in the limit *ω* → 0. In this limit temperature at outer boundary of the system is close to *A*_0_ for infinitely increasing amount of time, as is in fact temperature throughout the whole medium as the system thermal relaxation time is finite. Therefore, critical conditions must be essentially determined by the maximum temperature of the system during period of oscillation, and that is controlled by the value of the amplitude parameter *A*_0_. Dependence on the frequency must disappear in the limit *ω* → 0 as the explosion induction period (which is finite) becomes much shorter than the time which the system spends near its maximum temperature value *A*_0_.

For the larger values of oscillation frequency the behaviour of the critical boundary is explained as follows. Again, thermal stability of the system is effectively determined by its behaviour at the temperatures close to the maximum (through oscillation cycle). If this maximum temperature is fixed, then thermal explosion induction period (at this temperature) becomes larger than the period of oscillations as the frequency of the latter increases. Therefore, along the critical boundary, oscillation amplitude must increase with the increase of frequency. Constant rate of increase suggests that along the critical boundary (at least in the range of frequencies considered) product of the maximum oscillation temperature and the time over which ambient temperature stays close to this maximum remains nearly constant. The latter time is inversely proportional to the frequency of oscillations.

### Boundary condition of the second kind

In the case of Newtonian heat exchange with surroundings an additional parameter, Biot number *Bi*, appears in the problem formulation. Influence of the three parameters *δ*, *Bi* and *Ar* is illustrated in Figs 3, 4, 5 where a pair of the parameters is fixed and remaining parameter is allowed to vary.

First the influence of the Frank-Kamenetskii parameter with the parameters *Ar* and *Bi* being fixed is examined.

The dependencies in Fig. 3 are essentially linear, with critical amplitude decreasing with increase in *δ* (at fixed frequency *ω*). The latter trend is expected as with increasing Frank-Kamenetskii parameter the state of the system approaches the critical one at steady ambient conditions.

Further, Fig. 4 reveals that dependence of the critical conditions on Biot number is in fact quite weak.

Finally, the influence of Arrhenius number is demonstrated in Fig. 5. The trends are very similar to what is observed for the boundary conditions of the first kind (Fig. 2), although the critical amplitude is generally somewhat higher.

Generally, the trends observed in Figs 3, 4, 5 have the same qualitative explanation as in the case of the boundary conditions of the first kind.

### Unsteady development of thermal explosion

It is quite instructive to observe development of thermal explosion in time (for supercritical values of controlling parameters).

First, consider temperature distributions inside the slab. It turns out that at low frequencies temperature oscillation frequency at any point in the medium follows the frequency imposed at the boundary quite closely. Consequently, at low frequencies of oscillations, temperature profiles remain monotonic across the media.

**Figure 6:**
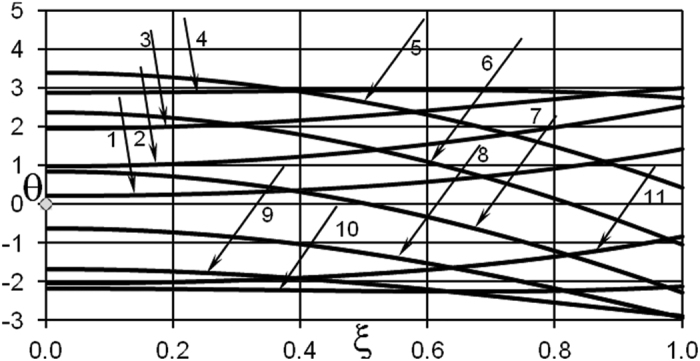
Temperature profiles inside the medium. *δ* = 0.2; *Ar* = 0.04; *ω* =10.0; *A*_0_ = 3.0 BC of the first kind. 1 − *τ* = 0.05; 2 − *τ* = 0.1; 3 − *τ* = 0.15; 4 − *τ* = 0.2; 5 − *τ* = 0.3; 6 − *τ* = 0.35; 7 − *τ* = 0.4; 8 − *τ* = 0.45; 9 − *τ* = 0.5; 10 − *τ* = 0.55 ; 11 − *τ* = 0.6.

However, a deviation from this simple pattern develops as frequency of oscillations grows. Temperatures at locations deep inside the media lag behind those at the external boundary. This is evidenced, for example, by the profiles 1, 2, 3 in Fig. 6. Such behaviour leads to appearance of non-monotonic profiles, such as the profiles 4 and 10 shown in Fig. 6.

**Figure 7:**
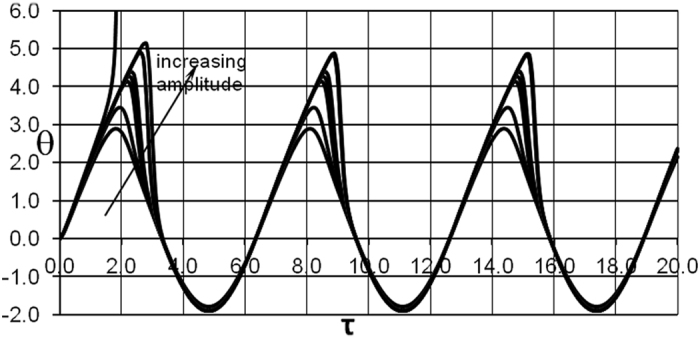
Temperature time history at *ξ* = 0. *δ* = 0.2; *Ar* = 0.04; *ω* *=* 1.0; *A*_0_ = 1.8 BC of the first kind. Arrow points out in the direction of amplitude increase for the following values: *A*_0_ = 1.8; 1.9; 1.93; 1.931; 1.9315; 1.9318; 1.931802. Thermal runaway curve corresponds to *A*_0_ = 2.0.

Temperature time history can be illustrated taking a fixed point inside the slab, for example the symmetry plane *ξ* = 0 where the temperature is normally maximal in the case of steady ambient conditions. With oscillating boundary conditions, this is obviously not the case anymore as evident from Fig. 6. Temperature oscillations at the centreplane of the slab are shown in Fig. 7.

It is interesting to observe behaviour of the temperature for conditions below critical. These are basically oscillations, and for the low amplitude, say *A*_0_ = 1.8, the curves are quite symmetrical. The positive branch of the curve have however stronger maximum amplitude, close to ≈3 compared to the negative semi-period of oscillation where the absolute value of the maximum amplitude reaches ≈2. This is obviously caused by contribution from the chemical term during positive semi-period of oscillation. This contribution becomes much more pronounced when amplitude increases up to *A*_0_ = 1.931802. Not only amplitude increases considerably during the positive semi-period of oscillation, but the peak of oscillation also exhibits noticeable delay compared to the curves corresponding to lower frequencies. Thermal runaway in the case of parameters presented in Fig. 7 occurs, upon further increase of the imposed amplitude, early in the process development, that is before completion of the first period of oscillations corresponding to subcritical values of the amplitude.

**Figure 8:**
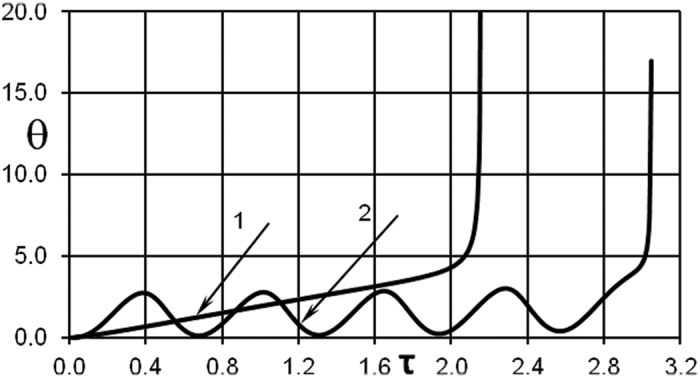
Two types of possible thermal explosion development. Temperature time history at *ξ* = 0. Curve 1 - BC of the second kind. *δ* = 0.2; *Bi* = 15; *Ar* = 0.04; *ω* *=* 1.0; *A*_0_ = 1.8 Curve 2 - BC of the first kind. *δ* = 0.6; *Ar* = 0; *ω* *=* 10.0; *A*_0_ = 2.8.

The case presented in Fig. 7 is not however the only type of explosion development that is possible. Data presented in Fig. 8 demonstrates that thermal runaway may in fact occur after a number of oscillations (curve 2, Fig. 8). The scenario in this case is that the peak amplitudes grow slightly from one oscillation period to another, and this is sufficient for the thermal explosion eventually to occur. This example manifests the new finding that oscillations are possible during induction period of the thermal explosion. This is a new (in the one-dimensional unsteady problem formulation) type of the system behaviour identified in media susceptible to thermal explosion.

Figure 8 summarizes both types of possible thermal explosion scenarios.

Data presented in Figs 1, 2, 3, 4, 5, 6, 7, 8 summarizes influence of all the controlling parameters in the basic formulation of thermal explosion problem with oscillating ambient conditions.

It is instructive to make estimations of the values of typical dimensional parameters involved.

It can be derived from the kinetic data for various gaseous and solid fuels^26^,^27^,^28^ that at least the range of amplitudes and frequencies 0 ≤ *A*_0_ ≤ ≈ 2; 0 ≤ *ω* ≤ ≈ 2 is practically relevant as it conforms with variations of the ambient temperature over ≈35° interval, and the dimensional frequencies from ≈6.10^−5^ *s*^−1^ to ≈6.10^−7^ *s*^−1^. The above temperature range is realistic for daily or yearly temperature variations. Further, the higher frequency value shown above corresponds (approximately) to daily oscillations, while the lower value of that range corresponds to yearly variations.

Daily or yearly ambient temperature variations are highly relevant for the problems involving accidental ignition and subsequent fires of solid materials stored or transported in significant quantities. Such are the fires occurring in large piles of refuse-derived or biosolid fuels, food grains, coal, bagasse, compost, energetic materials involved in military applications, and similar fuels^1^,^2^,^3^,^4^,^5^,^6^,^7^,^8^,^25^.

The above range of critical parameters (*A*_0_, *ω*) has been estimated based on quite limited kinetic data. Since the range of fuels that may undergo thermal explosion is quite versatile, much wider range of (*A*_0_, *ω*) values is likely to turn out to be practically relevant if comprehensive evaluation of kinetic data is conducted.

Therefore, thermal explosion scenarios considered in the present paper may well occur in real combustion systems. Development of explosion in practical situations may have quite complicated nature, such as a runaway following a number of temperature oscillations.

Interpretation of results with regard to fire risk is as follows. Lower oscillation frequencies require lower amplitude for explosion development as the system stays longer close to the maximum temperature of the oscillations. This has a very important practical implication as oscillations with low frequencies and amplitudes are most difficult to detect.

However, the most important finding of the study emphasizing the risk of oscillating conditions is the fact that oscillations may transfer the system from the state of thermal equilibrium to explosion conditions. At *ω* = 0 (no oscillations) parameters of the system are below critical in all the Figs 1, 2, 3, 4, 5, yet the explosion become possible if oscillations develop.

## Conclusions

The present paper considers the problem of thermal explosion development in oscillating ambient conditions. This is a generalization of a classical thermal explosion problem. Two physically meaningful types of boundary conditions are considered, and all the parameters controlling the system behaviour identified.

Explosion critical conditions are presented as dependencies of oscillation amplitude on oscillation frequency with all the other parameters fixed. These dependencies, that are critical boundaries, demonstrate oscillation amplitude increasing monotonically with the increase of oscillation frequency. In most cases (in the range of parameters investigated) the growth of amplitude with frequency along the critical boundary is very close to linear, except for very low frequencies. In the limit of oscillation frequency approaching zero, critical oscillation amplitude becomes independent of the frequency. Insight into such behaviour of critical conditions is provided by comparing characteristic time scale of the system being close to its maximum temperature during oscillations with the explosion induction period of the system under steady ambient conditions, fixed at the same maximum temperature.

Unsteady development of thermal explosion is investigated. It is discovered, for the first time in the system with spatially distributed parameters, that along with well known monotonic temperature rise during induction stage of the explosion, there exist regimes where temperature oscillations occur during induction period. Behaviour of temperature profiles in the media during thermal explosion development is investigated. It is shown that non-monotonic profiles develop across the medium at high frequencies of ambient temperature oscillations imposed at the external boundary of the system.

Practical applications of the presented formulation of the thermal explosion problem are discussed. It is demonstrated that the formulation is of practical interest for the spontaneous combustion problems where daily or seasonal temperature variations are important. These are the problems related to accidental fires occurring in storages of various solid materials, well documented in the literature.

A range of parameters relevant for such practical applications is conservatively estimated based on available kinetic data. This range is likely to widen if more extensive study of properties of various solid materials is conducted. Calculations presented in the paper cover this practically important range of parameters, therefore the major effects reported in the study (transition from stable to unstable thermal behaviour of the system upon development of oscillations in boundary conditions, oscillations of the temperature of the system during induction period of thermal explosion) may well occur in practical scenarios leading to solid fuel autoignition and development of accidental fires.

## Additional Information

**How to cite this article**: Novozhilov, V. Thermal explosion in oscillating ambient conditions. *Sci. Rep.* **6**, 29730; doi: 10.1038/srep29730 (2016).
